# The effect of pain management group on chronic pain and pain related co-morbidities and symptoms. A stepped-wedge cluster randomized controlled trial. A study protocol

**DOI:** 10.1016/j.conctc.2020.100577

**Published:** 2020-06-18

**Authors:** Marjatta Reilimo, Leena Kaila-Kangas, Rahman Shiri, Marjukka Laurola, Helena Miranda

**Affiliations:** a Occupational Health Helsinki, Helsinginkatu 24, 00101, Helsinki, Finland; b Work Ability and Working Careers, Finnish Institute of Occupational Health, P.O Box 40, FI-00032, Helsinki, Finland

**Keywords:** Pain management, Chronic pain, Self-efficacy, Health services, Occupational health

## Abstract

**Introduction:**

In primary care settings, pain-management group therapy is a tool potentially cost-effective but very much underused.

**Methods:**

Our purpose here is to provide useful scientific information on the effect of pain-management group participation on chronic pain and pain-related co-morbidities and symptoms, as well as practical information for primary and occupational health services to initiate pain-management group activity.

This study will be carried out at primary care Occupational Health Helsinki (Helsinki city employees' occupational health services), with the Finnish Institute of Occupational Health as the research partner.

This is a stepped-wedge cluster randomized controlled trial among both male and female municipal employees aged 18 to 65, all of whom had visited an occupational doctor, nurse, psychologist, or physiotherapist because of any chronic pain unrelated to malignant disease. An additional inclusion criterion is work disability risk being elevated, based on a short screening questionnaire (modified Örebro questionnaire). Each participant and each interviewer will be blinded at randomization.

Three groups, 10 subjects in each, begin directly after recruitment with 6 weekly 2-h meetings and a follow-up meeting 6 months later. Three waiting-list groups begin 4 months later. Subjects complete self-administered questionnaires before and after the sixth meetings, also 6 months later. Primary outcomes are pain intensity, current work ability, pain self-efficacy, fear-avoidance beliefs, chronic pain acceptance, depressive symptoms, sleep problems, sickness absence days, and number of occupational health care contacts from OH's medical records.

**Results:**

We will publish our results in a peer-reviewed scientific journals.

## 1 Introduction

In 2016, low-back pain and migraine were established to be in the top ten of diseases or injuries, causing years lived with disability (YLD) in countries and territories numbering 195 [1]. Among all citizens in European countries and Israel, those reporting moderate or severe chronic pain as lasting at least 6 months, experienced in the previous month, and at least twice a week, has ranged in Europe from 12% to 30%. Pain prevalence by this definition is highest in Norway, Poland, and Italy, and lowest in Spain, Ireland, and the UK [2]. The prevalence of any chronic pain condition, surveyed in 10 developed and 7 developing countries, was higher in women than in men, and the incidence of chronic pain was found to increase with age [5]. In New Zealand, the prevalence of chronic pain is 16.9% [3]. In Finland, the chronic pain prevalence was reported in 2006 to be 19% [2], with approximately one million Finnish chronic pain sufferers, of whom the majority were actively participating in working life [4]. According to the 2006 survey in Europe and Israel Finns were more often absent from work due to chronic pain than are other Europeans, up to three times as often as in Sweden or in the UK [2]. Over 30% of Finns' working days are lost due to pain-related disorders, mainly due to joint pain, neck, arm, and back pain. Furthermore, recurring shoulder and neck pain has burdened more than 40% of working people on Finland [6]. Moreover, the number of disability pensions in Finland due to musculoskeletal disorders has been very high, i.e., about 30% of annually granted disability pensions in 2015 [7].

Chronic pain rarely occurs alone and therefore we chose our outcomes based on the earlier chronic pain research results. Common co-morbidities are depression and sleep problems [[8], [9], [10]]. Those who sleep poorly have reported significantly higher scores on the Beck Depression Inventory (BDI) and higher baseline pain intensity ratings [11]. Moreover, a Chinese study has found obesity to be associated with chronic pain [12]. In a Finnish population-based study among working people, half of the subjects experienced either widespread pain, depression, or sleep disturbance [13], with one-fourth having at least two of these symptoms concurrently. Workers with co-morbid widespread pain, depression, or sleep problems had a 10-fold higher risk for reduced work ability and sickness absences, and their visits to a physician were 4–5 times as high as among those without these symptoms [14]. When pain is associated with inability to work, both women and men are more likely to experience pain in several parts of the body, to smoke, to have a lower level of education, and to be obese [15].

Finland's Current Care Guidelines of chronic pain management recommend primarily non-pharmacological treatment [16,17]. The most useful types of such treatment, based on increasing evidence, include cognitive-behavioral therapy (CBT), exercise, mindfulness-meditation, yoga, acupuncture, and music listening, as well as treatment combining these methods [18].

One cost-effective way to deliver this treatment is via group activity. In several studies, pain management group therapy with a cognitive-behavioral framework had a significant effect on chronic pain [[19], [20], [21], [22]]. CBT often involves learning-attention management, in which attention is moved away from the pain to more meaningful things. Attention management therapy for six weeks has reduced chronic-pain-related anxiety and hyper-arousal as well as reducing the impact of chronic pain on everyday life [23]. CBT alone and in combination with physical therapy has significantly reduced long-term sickness absence and the use of health services among back- and neck-pain patients, when compared to a control group who received only clinical examinations and guidance [19].

Chronic pain patients' pain is usually very high. In neck pain research, mean pain intensity (VAS) of 72 patients was 6.3 cm [24]. Pain experiencing can be affected by fear of the harmful effects of pain, such as pain itself and fear of injury. In addition, patients' pain experience may be widely influenced by factors such as fear, distress and false beliefs of the nature of pain and the outcome of treatment [25]. One meta-analysis concluded that correlation between pain-related fear and disability was 0.50, which makes the connection quite significant [26]. Self-efficacy is protective, whereas fear-avoidance belief is a risk factor for quality of life [27,28]. Health care professionals have been poor at identifying psychological risk factors for low back pain (LBP) like life crises, fear, anxiety and depression [29]. Lethem et al. generated the fear-avoidance model to explain why some individuals having musculoskeletal pain, became chronic, especially lower back pain. The centerpiece of that model is the fear of pain in lifting, bending, or working. Avoidance of such activities exacerbates the fear of pain [30,31].

La Chapelle, Lavoie, and Boudreau showed six different phases in the process of accepting pain [32]. First, the patient feels a need for help, after which the found help usually leads to receiving a diagnosis, then health professionals usually tell the patient a diagnosis, and after that the patient realizes that there is no sudden cure. After all these phases, acceptance leads to understanding that things could be worse, which leads to redefining normal, and after that, acceptance may be seen as an ongoing daily process. Factors that increase pain acceptance can include getting a diagnosis, good social support, educating yourself and others close to you about chronic pain, caring for yourself and being merciful to yourself. Factors preventing pain acceptance can be the struggle to preserve the identity that prevailed before chronic pain, negative relationships, other people not accepting chronic pain, the unspoken messages and spoken messages of other people that a person who looks so healthy cannot be so painful [32]. Pain acceptance has been found to relate to positive mood. Furthermore, when pain acceptance increases the levels of positive feelings, it reduces the negative mood [33].

Physical symptoms often occur without physical illness; this is called “somatization,” when psychological factors cause a symptom. The somatization tendency makes it more likely that one will seek medical help [34]. Somatization may amplify transient pain sensations, making them more persistent [35]. High self-efficacy attenuates the association between perceived pain and somatization and researchers suggest that clinicians should encourage especially those pain patients' self-efficacy, who are predisposed to somatization [36].

The aim of this study is to examine the effectiveness of pain-management group participation on pain intensity and associated disability, pain self-efficacy, fear-avoidance beliefs, acceptance of chronic pain, and co-occurrent mental or sleep problems in patients with chronic pain as well as their perceived ability to work, their sickness absence (SA) due to pain, and their use of health services.

## 2 Methods

### 2.1 Participants and recruitment

The study base comprises the employees of the city of Helsinki, the country's largest employer. The city of Helsinki has its own occupational health services (OHS), which provides OH services for more than 40 000 employees in 800 occupations of 30 industrial fields. The average age of employees in the city of Helsinki in 2014 was 46, the largest age-group in the city being 50–59. The youngest employees were 19 years old and the oldest employees of the city of Helsinki were 65 [37]. We therefore decide to include employees aged 18–65 years. Systematic review stated that most often the exclusion criteria's were psychological conditions, malignancy, recent or scheduled surgery, pregnancy and trauma [38]. Consistently we will exclude severe diseases such as malignancies, which would lead possibly to missed meetings and lost follow-up, and also we exclude a disease such as severe mental illness, which could bias results [39].

Inclusion criteria

• 18- to 65-year-old Helsinki city employee
• Chronic pain (lasting) for 3 months or longer
• Suitable for group activity, by being willing to share own thoughts with other group members
• Work disability risk being elevated, based on a short screening questionnaire (modified Örebro questionnaire). Screening survey points for work disability ≥50/100

Exclusion criteria

• Malignant disease such as cancer or severe mental disease
• Participating in another pain-management group
• Experiencing a major psychological or physical life crisis
• Being at no elevated risk for work disability, Screening survey (modified Örebro questionnaire) points <50/100

### 2.2 Sample size calculation

In this study we have 9 outcomes. In the presence of several outcomes, sample size should be calculated for the outcome with the smallest difference in effect size before and after [40]. Chronic pain patients' pain is usually high, even 7 or higher, and pain often remain with no change in clinical treatment studies. Minimal cut-off change for pain has been detected in previous studies. Salaffi et al., 2004 [41] stated that minimal cut-off point measure of Numeric Rating Scale of NRS pain is −1.0 cm. When pain is 7.5 before intervention, and after intervention 6.5, the effect size is 13%. The difference in pain acceptance and fear-avoidance beliefs may even be larger than 15% pre- and post-intervention [41]. By using stepped wedge analysis on the study design matrix (6 groups with 4 observations of each group) and with 15% detectable minimum difference of the outcome (chronic pain, mean 7.14 in a scale from 0 to 10) we calculated the minimum group sample size to be 10 (when a-error is 0.05 and b-error 0.80) and total number of participants to be 60 [42].

### 2.3 Randomization

Occupational health nurses, doctors, and psychologists and physiotherapists are responsible for recruiting 60 mainly Finnish study participants and know well the participants’ medical history and with the participants go through all inclusion and exclusion criteria. Meta-analysis by Bernardy et al., in 2010 involved whether CBT has any effect on fibromyalgia symptoms. The median number of patients with CBT was 40, with group size ranging from 7 to 64. Of the 527 patients in the CBT groups, 81% and in the control groups 75% completed therapy [20]. Consistently, we estimate those, who will take part in our study as ranging from 75% to 81%. Choice of the final sample size is influenced also by its being unethical to have a large sample if the benefit of treatment is unknown. Final sample size is also influenced by the adequacy of financial resources. We will ask for voluntary group leaders among its personnel: psychologists, nurses, and physicians. Moreover, we will recruit one pain nurse from the hospital pain clinic. Three tutor pairs comprise group leaders. In each patient group will be 10 participants, the recommended group size (8–10 participants) for the tutor to be able to work with every participant during the 2-h meeting. Six groups with 10 participants each is estimated to provide sufficient data for the statistical analyses, with no pilot study data.

Since this intervention includes pain psychotherapy (CBT, mindfulness, attendance, and commitment therapy ACT) with topics about chronic pain differing in every meeting, including homework, increasing the sample size to account for dropout is not recommendable. With increasing sample size, new participants would not take part in all group meetings, which could weaken peer support and reliability and the validity of research results. Subjects who fulfill the inclusion criteria (Table 1) and express an interest in participating in the study will be individually interviewed (H.M, M.R). In this interview, inclusion and exclusion criteria will be topics asked about, in person. All will be informed about the study, and provide their written informed consent including their participation as being voluntary. They will receive information stating that during the study, each subject is able to use occupational health services as before. During the recruitment phase, the subjects fill out the short screening questionnaire (Scr), the modified Örebro questionnaire.

**Table 1:** Condensed outline of meeting content.

	
First meeting:	• Tutor introduction, goals, rules, practical issues • Participant introduction in pairs, brief description of the symptoms of pain, current work ability, motivation to participate, expectations • Discussion on pain management tools already in use • Homework: “Foreword, *A New Understanding of Pain, My Story*” (pages 1–39) in book Rethinking pain
Second meeting:	• Short Relaxation/Mindfulness Practice before every meeting • Theme of the meeting: new knowledge on pain, mechanisms, differences between acute and chronic pain, individuality of pain, reality of pain, role of the brain and central nervous system • Discussion on pain mechanisms • Discussion about homework Homework: “Fostering Sleep” (pp.54–61), “Doing What You Enjoy” (pp.80–102), “Be Aware of Presence” (pp.142–149), “Yoga” (pp.194–198) in book Rethinking pain
Third meeting:	• Themes of the meeting: sleep, mindfulness, meditation, yoga • Group work on 1. Doing pleasurable things will help improve pain management 2. How pain affects social life, and vice versa • Discussion of homework Homework: “Work” (pp.150–161) and Appendices 2 (pp.232–233), and 3 (pp.234–241), link to www.otakipuhaltuun.fi, link for awareness exercise for Orton's pain patient (duration: 10 min, by psychologist Esko Silen) https://www.youtube.com/watch?v=xczkxCdNYmQ
Fourth meeting:	• Theme of the meeting: work • Pair discussion: The importance of work in life. Objectives of work. Professional development. The future of working life. Supervisors and co-workers - hopes and expectations directed towards supervisors and co-workers. On sick leave or at work despite pain. What can be done at work to improve work ability. Pairs present their thoughts. Group discussion follows • Discussion of homework Homework: “Develop Positivity” (pp.62–71), “Touch and Be Touched” (pp.72-29), “Talk about Your Emotions” (pp.103–112), “Love” (pp.162–169), “Get a Pet” (pp.170–177)
Fifth meeting:	• Themes of the meeting: emotions, love, touch, sexuality, nurturing a positive attitude, having a pet • Discussion of homework Homework: “Nourish Yourself” (pp.113–127), “Exercise With Joy” (pp.128–141), “Manage Your Weight” (pp.178–187), “Quit Smoking” (pp.188–193), “Cultural Power” (pp.205–214) + Appendix 1 (p.230): “Things You Can Affect And How They Affect pain”.
Sixth meeting:	• Themes of the meeting: new tools to improve pain management, making one's pain management plan • Filling out the follow-up inquiry • Discussion of homework and pain management tools. • Homework: The pain management plan will be given for writing at home
Seventh meeting, 6-month follow-up, Eighth meeting 12-month follow-up for ABC groups	• Themes of the meeting: psychological flexibility, importance of training and awareness skills, work as rehabilitation • Updating the pain management plan

Both participants and interviewers are blinded for the randomization, and at the end of the interview participants are randomized to one of the six groups (A, B, C, D, E, F). In this stepped-wedge design, every participant knows that he or she will receive an intervention, and the researchers know that no one will be left without treatment, which is not the case in conventional RCT [43].

The request for pain-management group activity originates with the patients themselves. Occupational Health Helsinki has provided three other kinds of groups during the previous 5 years (groups for sleep problems, depression, and burnout). The practical framework for our pain-management group is based on the feedback from those groups (size, facilities, timing, length, language, visual tools). After discussion between the leadership of the city of Helsinki and Occupational Health Helsinki, the results of this study will be provided to the participants in the form of the result report (via e-mail) as well as articles in the media (OHC website, Twitter and such).

### 2.4 Study design

**Fig. 1:**
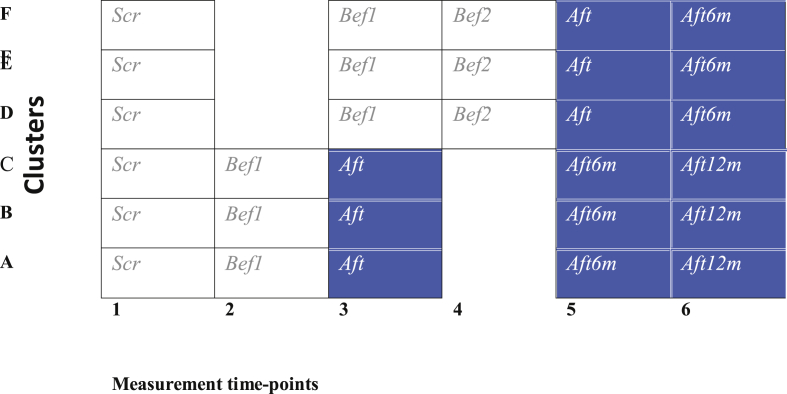
Study design of a stepped-wedge cluster randomized controlled trial. Shaded cells are intervention periods, and blank cells are control periods. This trial has six data-collection points. Surveys: Scr = screening, Bef1 = before intervention (1st time), Bef2 = before intervention (2nd time), Aft = immediately after intervention, Aft6m = 6 months after intervention, Aft12 m = 12 months after intervention, for only half of the clusters.

This study is a randomized controlled trial with a stepped-wedge design, where all clusters and participants in clusters receive intervention eventually in random order [43] (Fig. 1). Participants are randomized to either a group starting intervention within 1–3 weeks after the recruitment, or to a waiting-list group. The waiting-list group starts the intervention after the first group has completed the intervention, i.e., after approximately 4 months. Three groups (A, B, C) with 10 subjects each start the intervention immediately, and three groups (D, E, F) with 10 subjects each are on the waiting list.

The participants’ own OH nurses and doctors are informed about the study and are responsible for the participants' medical care as usual, if needed. The researchers (H.M, M.R.) are not involved in treating the study participants during the study, to prevent any influence on the results. No restrictions exist in initiating or changing the pain medication or other treatment modalities or examinations during the study.

### 2.5 Timetable

**Fig. 2:**
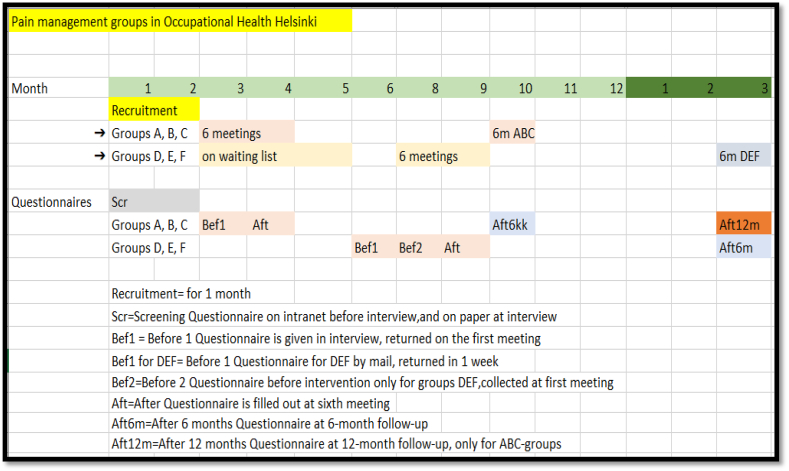
Timetable for the intervention.

Recruitment lasts for 1 month, with the Screening (Scr) Questionnaire (modified Örebro questionnaire), filled out before the interview and during the interview. ABC groups start their six meetings one month after recruitment ends. The before (Bef)1 questionnaire is completed during the interview and returned at the first meeting. The After (Aft) questionnaire is filled out during the sixth meeting. DEF groups are on the waiting list and get their Bef1 questionnaire by mail, while the first three groups (ABC) are in the intervention. Bef1 is returned in one week. DEF start the intervention, six meetings 4 months after the first intervention. The Bef2 questionnaire is sent by mail to the DEF groups before intervention and collected on the first meeting day. The Aft questionnaire is completed at the end of the sixth meeting, and participants are advised to book time for their own occupational health nurse if a group member wishes support in completing the pain management plan. Aft6m is filled out at the 6-month meeting. The Aft12 m is completed in the meeting 12 months after intervention; the Aft12 m meeting is arranged only for the ABC groups (Fig. 2).

### 2.6 Intervention

The pain management groups are led by occupational health professionals (Helsinki city's OH personnel) who voluntarily participate in this study. The group leaders are experienced group tutors, and they will have mastered the biopsychosocial and cognitive approach. An experienced pain psychologist is hired as a mentor for the group tutors. The group meetings are once a week, after work, lasting 2 h with a 15-min coffee break for 6 weeks. During the intervention, participants will read some chapters from the book *Ota kipu haltuun* (Rethinking pain) by Helena Miranda, as homework [44] (Table 1). The follow-up meeting is 6 months after the sixth meeting. The second follow-up meeting is 12 months after the sixth meeting, only for A, B, and C groups. The content of the meetings is in Table 1.

### 2.7 Data collection

Data collection can be seen in Table 2. Variables. During the recruitment, a short screening questionnaire (Scr), a slightly modified version of the validated Örebro Short Questionnaire is filled out [45]. A question as to the pain lasting more than 3 months is not calculated, it is one of the inclusion criteria. The sum score is calculated based on 10 questions, each earning 0–10 points: pain areas, (head, neck-trapezius, shoulder/upper arm, elbow or lower arm, wrist/hand, upper back, lower back, hip/thigh, knee/shin, ankle/foot-pain), pain intensity, anxiety, depressive symptoms, being able to do light work for an hour, sleep, expected risk of pain remaining persistent, self-perceived ability to work within the next 3 months, two items on fear-avoidance: “I should stop what I am doing until the pain decreases” and “I should not do normal activities or work when I feel pain”. Maximum sum is 100 points.

**Table 2:** Variables.

Variables		Questionnaires			
		Scr	Bef1, Bef2	Aft	Aft 6 m, Aft 12 m
*Outcomes*					
	Pain intensity, 1 item (0 = no pain, 10 = the worst possible pain)	x	x	x	x
	Current work ability, 1 item (0 = totally disabled, 10 = the ability to work at its best)		x	x	x
	Pain self-efficacy, 10 items (0 = not at all confident, 6 = completely confident)		x	x	x
	Chronic pain acceptance, 8 items (0 = totally disagree, 6 = totally agree)		x	x	x
	Depressive symptoms, 1 item (0 = not at all, 10 = extremely much)	x	x	x	x
	Sleep problems, I can sleep at night (0 = I can do it despite pain, 10 = I can't do it because of the pain problem)	x	x	x	x
	Sleep problems, days slept well during the past week (0–7)		x	x	x
	Sickness-absence days (6 months before and after intervention)				
	Health care contacts, number of contacts (6 months before and after intervention)				
*Pain-related variables*	Pain areas, 10 areas (0 = no, 1 = yes)	x	x	x	x
	Pain-related fear-avoidance, 2 items (0 = totally disagree, 10 = totally agree)	x	x	x	x
	Fear of movement, 3 items (0 = totally disagree, 6 = totally agree)		x	x	x
	Self-perceived risk of current pain becoming persistent (0 = no risk, 10 = very large) risk)	x	x	x	x
	Use of painkillers during the previous week, 3 items (0 = none, 1 = few times a week, 2 = every day) Anti-inflammatory drug or Paracetamol Strong analgesic, opiate, medications for chronic pain		x	x	x
	Other medication (open question)		x	x	x
	Non-pharmaceutical pain management tools (open question)		x	x	x
*Work ability variables*	Current ability to do light work for an hour (0 = can do despite pain, 10 = can't do at all because of pain)	x	x	x	x
	Self-estimated work ability in 3 months, 2 items (0 = very large chance, 10 = no chance)	x	x	x	x
	Sickness-absence days during the past 30 days (0–30)		x	x	x
	Sickness-absence days for pain during the past 30 days (0–30)		x	x	x
*Other*	Days enjoying life during the past week (0–7)		x	x	x
	Days feeling active and energetic during the past week (0–7)		x	x	x
	Days feeling tense or restless during the past week (0–7)		x	x	x
*Covariates*	Age	x			
	Gender	x			
	Occupational title		x		
	Height		x	x	x
	Weight		x	x	x
	Physical exercise, number of times per week		x	x	x
	Kind of sport/sports, open question		x	x	x
	Chronic disease, (0 = no, 1 = yes)		x	x	x
	Chronic disease, if yes, which, open question		x	x	x
	Smoking, (0 = no, 1 = no, quit, 2 = yes)		x	x	x
	Quit smoking, years smoked		x	x	x
*Benefits of*	Usefulness of mindfulness practice (0 = not at all useful, 10 = very useful)			x	
	Usefulness of peer group for different issues, 17 items (0 = not at all helpful, 6 = very helpful)			x	
	Ranking importance of features of peer group (1 = most helpful, 2 = next most helpful, etc.) 5 items			x	
	Benefit from participating in pain- management group (0 = not at all useful, 10 = very useful)				x

After the recruitment and interview, the baseline questionnaire (Bef1) is completed or subjects are given a 1-week response time. The waiting-list group fill out an additional questionnaire during the waiting-list period (Bef2). After intervention, the first follow-up questionnaire (Aft) is filled out, and the second follow-up questionnaire (Aft6m) at the 6-month follow-up group meeting (seventh meeting). The third follow-up questionnaire (Aft12 m) is sent via mail only for the first intervention group.

The content of the baseline and of the follow-up questionnaires is, to a large extent, the same. They contain the same 10 questions from the screening questionnaire, as well as questions on pain-related fear, pain self-efficacy, chronic pain acceptance, self-rated work ability, the number of sick-leave days due to any reason and due to pain, medication use for pain and for other purposes, non-pharmaceutical treatments in use, feeling energized, enjoying life, physical exercise, smoking, body weight and height, and chronic diseases. Sickness-absence data and use of occupational health services is collected from the OHS patient register 6 months before and 6 months after intervention.

### 2.8 Outcomes

Outcomes, variables, and covariates are listed in Table 2.

Outcomes are pain intensity, current work ability, pain self-efficacy, pain-related fear avoidance beliefs, chronic pain acceptance, depressive symptoms, sleep problems, sickness absence days and number of occupational health care contacts which are collected from OH's medical records.

### 2.9 Variables

Pain-related variables

Pain areas, self-perceived risk of current pain becoming persistent is inquired about as well as the use of painkillers, opioid use, and use of chronic-pain medication. Furthermore, non-pharmaceutical pain management tools are also asked about.

Work-ability variables

We measure with questionnaires current ability to do light work for an hour, work ability in the next 3 months, and self-reported sickness-absence days due to any reason and due to pain.

Other variables

Number of days during the prior week spent enjoying life, feeling active, or days feeling tense or restless.

3.0 Covariates

Age, gender, occupational title, height, weight, amount of physical exercise during the past week, what kind of sport, whether the participant has a chronic disease, and if yes, which chronic disease; other medications, whether the participant smokes and how many years has smoked.

### 2.10 Interpretation and variability of outcomes

The maximum sum on the Örebro Short Questionnaire is 100. When a sum score is ≥ 50, the result may predict a higher risk for future work disability [45] (Table 2 the Scr test). We compare the before-intervention sum to the after-sum to discover whether work disability has decreased.

Perceived work ability may predict other work-related outcomes such as sickness absence, retirement, and disability [46]. We compare perceived work ability before and after intervention to discover whether our pain management group has any effect on perceived work ability of chronic pain patients. Furthermore, we learn whether returning to work or continuing working with one's normal duties in 3 months improved after intervention.

According to the literature, the pain visual analogical scale (VAS) gives the highest scores for some chronic pain patients before dinner or at bedtime and for other patients before breakfast or at lunch. Those with the greatest pain in the afternoon and at bedtime are more likely to experience widespread pain and difficulty in sleeping as well as making increased use of health services [47]. In other studies, VAS has not been significantly influenced by interventions. Here, we compare VAS before and after intervention to find whether participating in our pain management group reduces VAS.

McCracken defines acceptance of chronic pain as "a pattern of behavior in which activity is pursued in the presence of pain but without the limitations of pain or efforts to avoid or control pain" [48]. We estimate by comparing before and after values whether chronic pain acceptance has improved.

Co-morbidities commonly associated with chronic pain are depression and sleep problems. Depression can reduce acceptance of chronic pain and worsen pain [8] and may worsen chronic pain [10]. We compare before and after value for depression to interpret whether intervention reduces depressive symptoms and sleep problems.

The cognitive-behavioral fear-avoidance model of chronic pain propose that pain-related fear contributes to the development and maintenance of pain-related disability [27]. Fear-avoidance beliefs and behavior are strongly connected to low-back pain and high fear avoidance belief and behavior worsen the prognosis and treatment outcome. Furthermore, health personnel tend to increase chronic pain patients' fear avoidance especially if they themselves have high fear avoidance belief themselves [49]. We compare fear-avoidance beliefs before and after intervention to learn whether our pain management group weakens fear avoidance beliefs.

Self-efficacy beliefs in those with chronic pain may relate to confidence in performing specific tasks or to confidence in coping with pain [50]. We compare self-efficacy before and after intervention and explore whether our pain management group increases self-efficacy.

### 2.11 Statistical analysis

The post-intervention period is compared with the pre-intervention (control) period. Linear mixed-effect models allow analysis of repeated measures data and the differences in the outcomes of interest between intervention and control periods. Intention-to-treat analyses are conducted, and time-effect and intra-cluster correlation coefficients reported. When the null hypothesis (i.e., no difference) is rejected, it is always possible to conclude, whatever the results of the study are, that there exists a difference but actually there is not (type-I error or false positive) [51]. Since the sample size is rather small, exact confidence interval for outcomes will be the choice to avoid statistical bias and false-positive or false-negative errors [51].

## 3 Discussion

Chronic pain is globally one of the most general health problems [2]. It causes a physical and emotional burden on society, with estimated costs of €200 billion a year in Europe, and $150 billion a year in the USA [2]. In Finland one-third of the disability pension applicants in 2010 had comorbid musculoskeletal and mental health disorders [13].

More comprehensive and systematic pain management tools than in current practice: those such as prescribed pain medication, sickness absence, or surgery, are necessary to better manage the burden of work disability related to chronic pain. In OHS, it is possible to give better support to work ability, since the OHS staff are familiar with working conditions. OHS personnel may connect with the employer and negotiate tailored working conditions that match the employee's ability to work. The purpose of occupational health care is to promote employees' work capacity and functioning and reduce the effects of pain on work ability.

The conceptual framework of this study lies in the biopsychosocial nature of pain, as well as in the principles of acceptance, and commitment therapy, CBT, and on relaxation techniques and awareness skills. Acceptance and commitment therapy may have an effect on pain, functioning, depression, pain acceptance, cognitive fusion, decentering, and on the action involved [32]. Systematic relaxation techniques in older patients undergoing abdominal surgery have showed statistically significant differences in pain, in anxiety, and in analgesic use [52]. Mindfulness seems to reduce pain preparation, possibly reducing nociceptive information. Like other cognitive factors that modulate pain, mindfulness meditation also affects the prefrontal cortex and cingulate gyrus, which control pain modulation [53]. Group participation can potentially enhance the patient's own self efficacy and feeling of control over one's own symptoms and disability [54].

We deduced, based on earlier studies, that one weekly meeting for 6 weeks and a 6-month follow-up meeting would be sufficient for this trial. These preceding studies included a meta-analysis of the efficacy of CBT for the fibromyalgia syndrome. In 14 trials, treatment time was 5–15 weeks, median 9, and median follow-up was 6 months, ranging from 2 to 48 months. As a result, CBT significantly improved self-efficacy and significantly reduced physician visits [20].

If the results of our study are favorable, this study will help health care personnel to choose those chronic pain sufferers who will benefit from this type of treatment, especially regarding the association between pain-related fear-avoidance and work ability. One American meta-analysis including 46 trials showed that pain-related fear represents an important role in the management of pain-related disability [27]. Moreover, in this study, subjects will receive updated information on pain and its effect on sleep, mood, functioning, and work ability as well as on various non-pharmaceutical pain management methods. Group participation may potentially enhance the patients’ own self efficacy and feeling of control over their own symptoms and disability [55].

Most importantly, this study can provide for employees with chronic pain much-needed peer support. Intervention studying peer-support for military veterans with chronic musculoskeletal pain improved their self-efficacy and pain centrality, and researchers suggest that peers are able to effectively convey pain self-management strategies to each other. Peer support helps in acceptance of chronic pain [55]. A peer-support group is a potential tool to provide social support, self-management skills, self-confidence, and acceptance. Since pain cannot effectively be prevented, those with chronic pain can be taught to better manage their pain, and to live a full and meaningful life despite pain. A good life often also means being able to work.

Success in recruiting enough participants is likely, because one-third of those working in Helsinki city suffer from chronic pain, and most visit the OH for their pain. Furthermore, all professional groups (doctors, nurses, physiotherapists, psychologists) recruit patients.

## 4 Limitation

The sample size is rather small and therefore creates some limitations. For example, small size prevents generalization of results. However, based on our study results, a more extensive multicenter intervention study will possibly be planned for various occupational health services in Finland with separate funding. We choose a randomized-controlled trial with a stepped-wedge design, which has a relevant design when we desire that intervention does more good than harm. It would therefore be unethical to prevent some participants from being involved in an intervention, as happens in conventional RCT [43].

Based on our results, a more extensive multicenter intervention study will possibly be carried out in various occupational health services in Finland with separate funding.

## Trial registration

The study is registered in Clinical Trials, a service of the U.S. National Institutes of Health under number 115395, https://clinicaltrials.gov/ct2/results?term=115395&Search=Search.

## Contributors

MR and HM are the main authors. HM, LKK, and ML contribute to study design. MR is the chief investigator. MR and HM will undertake the recruitment and perform the study. RS will contribute the data analysis, and all these authors contribute their interpretations. All authors have reviewed and approved the final protocol manuscript.

## Funding

This study is funded by the 10.13039/501100003128Finnish Work Environment Fund (grant number 115395).

## Compliance with ethical standards

The study has been performed in accordance with the Helsinki Declaration. Ethical approval has been granted by the Coordinating Committee of the Hospital District of Helsinki and Uusimaa. All procedures performed in studies involving human participants are in accordance with the ethical standards of the Coordinating Committee of the Hospital District of Helsinki and Uusimaa and with the 1964 Helsinki declaration and its later amendments or comparable ethical standards.

## Data sharing

Results of the primary study will be submitted to a peer-reviewed journal. After publication of results, data requests can be submitted to the researchers.

## Declaration of competing interest

All the Authors confirm that there are no competing interests.
